# Electro-Optical Biosensor Based on Embedded Double-Monolayer of Graphene Capacitor in Polymer Technology

**DOI:** 10.3390/polym13203564

**Published:** 2021-10-15

**Authors:** Ary V. R. Portes, Ana J. L. Martins, Jesus Alvarez Guerrero, Mauricio M. Carvalho, Ferney O. Amaya-Fernandez, Lúcia A. M. Saito, Jhonattan C. Ramirez

**Affiliations:** 1 Department of Electronic Engineering, School of Engineering, Federal University of Minas Gerais (UFMG), Belo Horizonte 31270-901, MG, Brazil; aryv@ufmg.br (A.V.R.P.); anajlm@ufmg.br (A.J.L.M.); 2 Faculty of Engineering, Universidad Libre, Av. 4 No. 12N-81, Cúcuta 540008, Colombia; jesus.alvarezg@unilibre.edu.co; 3 Mackgraphe, Mackenzie Presbyterian University, São Paulo 01302-907, SP, Brazil; mauricio.moderno@outlook.com.br (M.M.C.); lucia.saito@mackenzie.br (L.A.M.S.); 4 Engineering School, Pontifical Bolivarian University, Circular 1 No. 70-01, Medellín 050031, Colombia; ferney.amaya@upb.edu.co

**Keywords:** polymeric photonic biosensors, electro-optical components, graphene-based capacitor, Fermi level control

## Abstract

In this work, we present an interferometric polymer-based electro-optical device, integrated with an embedded double-monolayer graphene capacitor for biosensing applications. An external voltage across the capacitor applies an electric field to the graphene layers modifying their surface charge density and the Fermi level position in these layers. This in turn changes the electro-optic properties of the graphene layers making absorption in the waveguide tunable with external voltages. Simultaneously, it is possible to appreciate that this phenomenon contributes to the maximization of the light-graphene interaction by evanescent wave in the sensing area. As a result, it is obtained large phase changes at the output of the interferometer, as a function of small variations in the refractive index in the cladding area, which significantly increasing the sensitivity of the device. The optimum interaction length obtained was 1.24 cm considering a cladding refractive index of 1.33. An absorption change of 129 dB/mm was demonstrated. This result combined with the photonic device based on polymer technology may enable a low-cost solution for biosensing applications in Point of Care (PoC) platform.

## 1. Introduction

Graphene has gained considerable attention when its unique properties have been reported in 2004 [1,2]. Since then, graphene has become a very versatile material due to its excellent electrical, optical and mechanical properties, and has been implemented in a wide range of applications [3,4,5,6]. These properties include widely tunable conductivity, dielectric constant, and real and imaginary parts of refractive index and enable electro-optic (EO) modulation [7,8,9,10].

Graphene can provide a high and very broadband optical absorption extending from visible to infrared range [11]. This absorption can be tuned over a very wide range by tuning the Fermi level using externally applied electric fields [12,13]. It also has very high carrier mobility, which can enable high speed operation. Graphene can also be incorporated into CMOS technology, enabling low cost and high functionality applications [14]. More recently, and due to the electrical characteristics previously described, graphene has been used for biosensing applications, where its high conductivity has been exploited for the generation of surface plasmon resonance [15,16,17], or for obtaining conventional electrochemical transducers, due to the high electron mobility that this material provides [18,19,20,21,22]. Additionally, because it is an organic material, it has been shown to be beneficial for this type of application, especially in its oxidized form [23,24], since conventional graphene, depending on the substrate used, can be perceived as hydrophobic [25], which can negatively impact.

Some features such as immunity to electromagnetic interference, high-speed operation, low power consumption, potential use in harsh environments, miniaturization, integration and multiplexing capability, mechanical stability, low-cost for fabrication, and real-time and label-less detection [26,27,28], have provided photonic biosensors a place of prominence before the scientific community, and not only thanks to the benefits previously listed, but also because this type of biosensors have demonstrated the best performance among all types of existing biosensors [29].

Most of the integrated photonic biosensors are based on silicon, due to the excellent optical characteristics that these materials present, demonstrating high sensitivities and a high reproducibility rate. However, despite all the benefits that silicon has, effects such as Two-Photon Absorption (TPA) and Free-Carrier Dispersion (FCD) can become evident, which would compromise the performance of said biosensor [30]. Therefore, devices based on polymeric materials are presented as a viable alternative, which despite having very low refractive indices and having shown low sensitivity, efforts are made by the scientific community to improve this scenario, compensating for the unfavorable optical characteristics of the material, with an efficient and reliable design [31,32,33]; our proposal goes in this direction. On the other hand, characteristics such as the biocompatibility of these materials enable simpler and more durable biofunctionalization processes, and its low production cost provides conditions for these devices to be massively produced for single-use [34], these are points to keep in mind when justifying its implementation.

The implementation of polymeric photonic devices, integrated with a graphene-based capacitive structure in a single device, will provide the conditions for obtaining an electro-optical biosensor with a wide evanescent field in the sensing area, and with low loss due to tunable absorption based on Fermi level control.

In this paper, we present a low-loss electro-optical polymer-based device, with an embedded double-layer graphene capacitor for biosensing applications. Our proposal demonstrates through theoretical analysis and numerical simulations the widening of the evanescent field and consequently, the increase in the sensitivity of the proposed device, due to the interaction generated between the electric field in the capacitive structure and the confined optical waveform. In this sense, a bulk sensitivity of 2828 nm/RIU and a surface sensitivity of 2021.85 nm/RIU were demonstrated for the proposed integrated photonic biosensor, both working with a gate voltage of $V_{g}$ = 20 V, being RIU the (Refractive Index Unit).

## 2. Simulation Modeling

An efficient use of graphene in these applications requires geometries that provide the correct interaction between the applied electrical fields in the graphene layers [35], and the optical waveform, for this reason, the need to obtain an optimized device for the proposed application is essential.

### 2.1. Materials and Methods

In this study, the Finite Element Method (FEM) was used for analyzing the sensor’s behavior by using commercial software COMSOL Multiphysics. The refractive index used in the core of the waveguide was 1.57 @ 800 nm wavelength, in the substrate it was 1.45 @ 800 nm wavelength, in the cladding of the sensor area it was varied between 1.33 and 1.38, and in the PMMA cladding it was 1.49 @ 800 nm.

**Figure 1:**
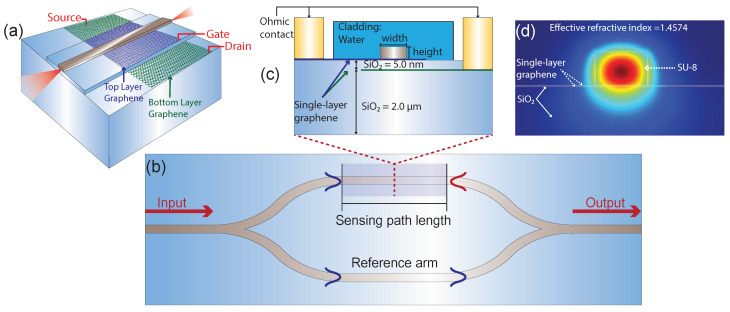
Design of the proposed polymeric sensor. (**a**) 3D view of a short section in the sensing area, where the interaction between the graphene-based capacitor and the polymeric waveguide, is presented. (**b**) Schematic of Mach-Zehnder interferometer biosensor. (**c**) Cross-section view of the sensing area, where the proposed device configuration is shown. (**d**) Mode distribution in SU-8 waveguide, interacting with the capacitive structure.

Our simulations were performed in two stages, first, the interaction between the projected waveguide and the graphene-based capacitor was analyzed using modal analysis. The number of elements used for this stage was 40,437. In addition, two physics were implemented to simulate the device. The first one, *electrostatics*, which is responsible for the generation of the surface current, through a potential applied on the ohmic contacts, as can be seen in Figure 1a,c. In this sense, the Gate terminal has a variable voltage $V_{g}$, while source terminal has a constant voltage of $V_{s}$ = 1 V and both Drain and Ground terminals has a constant voltage of 0 V.

In addition, the *electromagnetic waves* physics was implemented to generate the modal distribution at λ = 633 nm, λ = 800 nm, and λ = 1.2 μm for fundamental TE and TM modes, that would interact with the capacitive structure, whose composition based on graphene was modeled as a surface current boundary condition, which follows the Equation (1):

(1) j=σ·E

where *j* is the surface current density, σ is graphene conductivity and *E* is the electric field applied in the region, both by the voltage terminals and by the electromagnetic waves, demonstrating the perturbation generated to the optical mode by externally applied electric fields.

Similarly, in the second stage we use the same *electromagnetic waves* physics with 92,174 elements, to analyze the spectral response of the projected MZI with all the elements previously mentioned, presenting the expected phase change in the proposed photonic device, as a function of small variations in the sensing area.

### 2.2. Design of the Graphene-Based Capacitive Structure

The proposed simulation model is composed of a Mach Zehnder Interferometer (MZI) based on SU-8 polymer with 700 nm width and 450 nm height, on a Silicon Dioxide (SiO${}_{2}$) substrate, as illustrated in Figure 1a,b. The reference arm is unaltered, maintaining a PMMA cladding, on the other hand, the cladding in the sensor area is water, and a capacitive structure based on two monolayers of graphene separated by 5 nm of SiO${}_{2}$, was embedded below the polymer waveguide, as detailed in Figure 1c. In addition, each monolayer of graphene has a couple of ohmic contact gold terminals, Gate and Ground for the upper layer and Source and Drain for the lower layer. By applying an electrical voltage to these terminals, it is possible to control the Fermi level in this capacitor, modifying the optical mode in the waveguide, as presented in Figure 1d.

**Figure 2:**
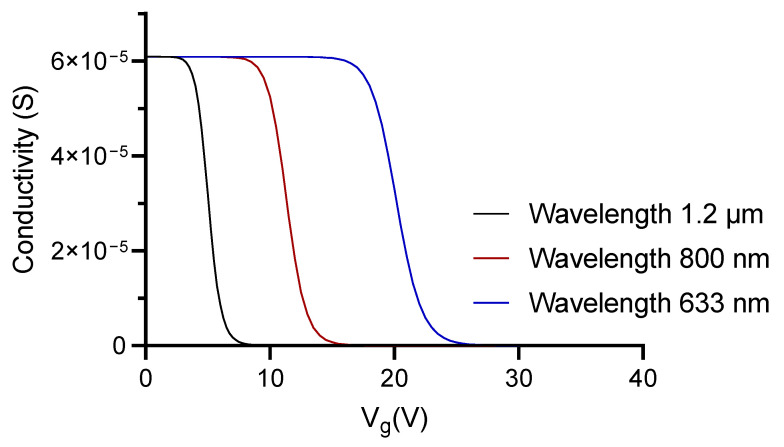
Graphene conductivity response as a function of $V_{g}$ applied voltage for 1.2 μm, 800 nm and 633 nm.

The integration of waveguides and graphene-based capacitors is a technology that has been widely exploited for electro-optical modulators, due to the tunable absorption of the graphene [8,9,36,37]. However, by using the same principle of operation for controlling graphene optical properties, it is possible to optimize the device, significantly improving the performance of biosensor components. Our hypothesis is based on the fact that the same electric field that controls the fermi energy in graphene, generates changes in the optical mode propagated inside of the optimized optical waveguide, altering the orientation and intensity of the electric field lines increasing the penetration depth of the evanescent field in the sensing area. Thus, it is possible to appreciate the dependence that exists of the permittivity of graphene with the variation of the Fermi energy (E${}_{f}$), as presented in Equation (2).

(2) $$\epsilon \left(\omega ,E_{f}\right)=1-\frac{i\sigma (\omega ,E_{f})}{\omega \epsilon_{0}\delta }$$

where δ is the graphene thickness, ω is the angular wave frequency, $\epsilon_{0}$ is the vacuum permittivity and σ is the graphene conductivity, which is a function of the angular wave frequency and the Fermi level [38]. By using the capacitive structure, graphene Fermi level and the applied voltage can be related by Equation (3).

(3) $$\mu_{c}=\hbar v_{f}\sqrt{\pi \eta |V_{g}|}$$

where $v_{f}$ is the Fermi velocity and $V_{g}$ is the gate voltage. The variable η is derived from the parallel-plate capacitor model, and it is defined by Equation (4), [39]:

(4) $$\eta =\frac{\epsilon_{0}\epsilon_{r}}{de}$$

where $\epsilon_{r}$ is the relative permittivity, *d* is the dielectric thickness between the capacitor plates and *e* is the electron charge. By using the Equation (4), the graphene conductivity was calculated for three different operating wavelengths, as presented in Figure 2. It is possible to observe in that figure that for the analyzed operating wavelengths, the conductivity of the graphene can be tuned from $6\times 10^{-5}$ S down to almost 0 S, setting 1 V in the Source-Drain interface, and increasing the Gate Voltage ($V_{g}$) from 0 V up to 40 V, consequently changing the permittivity of the graphene as well.

In Figure 2, it was shown that higher wavelengths have the conductivity transition at lower voltages, which is a key factor to reduce the power required. In order to reduce thermal effects in the polymeric device due to the high power required to tune the conductivity in the capacitive structure, and at the same time avoid the absorption of biological tissues in the infrared spectrum, we selected 800 nm as the operating wavelength for further studies [40]. It is important to highlight that some biological processes, especially related to imaging, are performed in IR wavelengths, however, the integrated photonic components are used mostly to carry out detection through antibody-antigen binding or, failing that, using DNA, RNA, etc. In these cases, the target protein is specifically detected on the device in the sensing area without the need for markers (label-free detection of diseases), this is our case, and when absorption by the analyzed protein is evidenced, we will have an abrupt reduction of the propagating light in the waveguide, along the sensing area, disabling the operation of the proposed interferometer, since the only contribution perceived at the output will be that of the control arm, this is observed in systems working in IR wavelengths.

**Figure 3:**
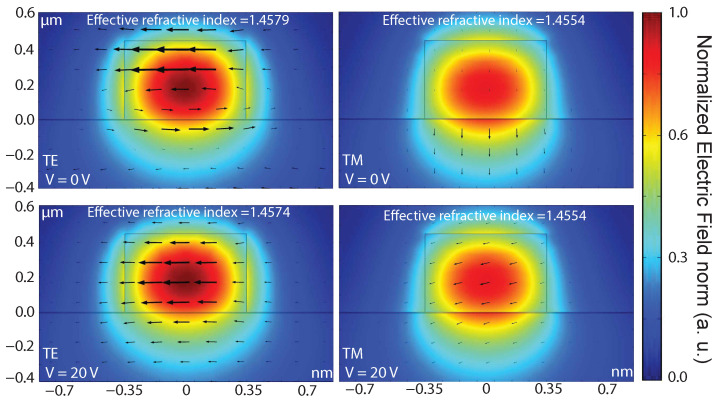
Electric field distribution at λ = 800 nm, with and without the impact generated by the variation of the Fermi level when $V_{g}=20$ V. In this figure it is possible to appreciate changes in the calculated field lines for the TE and TM modes, when 20 V at the gate is applied and a constant voltage of 1 V is maintained between source and drain, however, only the TE mode presents significant variation in the effective refractive index in the presence of the surface current generated by the electric field applied to the proposed capacitor.

According to Equations (2) and (3), it is possible to control the permittivity and the optical losses in graphene, modifying the confined optical mode collaterally, as can be seen in Figure 3. The electric field distribution in the TE and TM modes, were evaluated for 0 V and 20 V at the $V_{g}$, respectively. In this study it was observed that when applying 20 V in the $V_{g}$, the propagating TE mode shows a greater penetration depth of the evanescent wave in the cladding, reducing the interaction with the substrate previously identified in 0 V. This is evidenced by the reduction in the effective refractive index achieved. In the case of TM mode, in addition to the previously described changes, we have a slight modification in the orientation of the electric field lines, because of the applied voltage.

**Figure 4:**
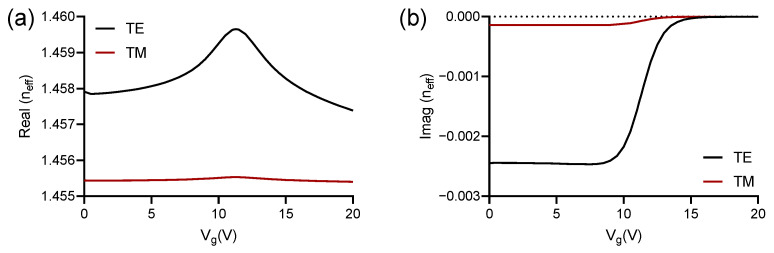
Variation of the (**a**) real and (**b**) imaginary parts of the effective refractive index for TE and TM modes as a function of the gate voltage ($V_{g}$) variation.

Next, we calculated the real and imaginary parts of the effective refractive index for the TE and TM modes, in order to choose the most suitable mode of operation for our study. In Figure 4a,b, we can see these results. As expected, the impact generated due to the voltage variation in $V_{g}$ is much greater in the propagation of the TE mode, which was demonstrated through complementary simulations, where the variation of the effective refractive index, as a function of small structural changes, was of the order of $10^{-3}$ in favor of it, which would strongly impact, when we talk about applications in biosensors. The results obtained through our numerical simulations can be seen in Figure 4.

### 2.3. Design of the Polymer-Based Component

**Figure 5:**
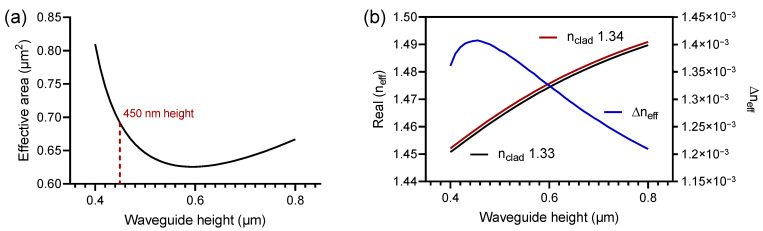
(**a**) Study of the behavior of the evanescent field, evaluating the effective area as a function of the height of the waveguide. (**b**) Analysis of the real part of the effective refractive index as a function of the height variation, for cladding values at 1.33 and 1.34, and the $\Delta n_{eff}$ as a function of the reference arm.

Achieving the best evanescent field in the sensing area while keeping the optical mode as confined as possible in the reference arm, is essential, Equation (5). In this sense, we computationally guided our photonic component, analyzing the effective area as a function of the height of the waveguide, having as a limitation the number of modes that we want to propagate and the minimum width that we can define for reasons of manufacturing restrictions. The results obtained can be seen in Figure 5.

(5) $$A_{eff}=\frac{{(\int \int \;|E\left(x,y\right)|}^{2}{dxdy)}^{2}}{{\;\int \int \;|E\left(x,y\right)|}^{4}dxdy}$$

Considering the Equation (5), and setting the width of the device in 700 nm after being optimized, based on manufacturing restrictions and required fundamental mode propagation, in Figure 5 the height of the device was varied between 400 nm and 800 nm in order to define the height at which the proposed device enhances its efficiency.

The expected result should guarantee a wide evanescent field in the sensing area allowing the largest possible $\Delta n_{eff}$ as a function of small variations in the refractive index in the cladding. In Figure 5a, it is possible to observe that the minimum effective area is achieved with a device with a height of 600 nm and the maximum one at 400 nm. This parabolic behavior is because in the region comprised between 400 nm and 600 nm height, the waveguide has a smaller size than the confined mode, for this reason as the height of the device is increased the evanescent field decreases, reducing the effective area. On the other hand, when the height is greater than 600 nm, we observe the increase of the confined optical mode accompanying the growth of the waveguide. According to the previous discussion, and the results presented in Figure 5b, where the $\Delta n_{eff}$ is shown as a function of small variations of the refractive index in the cladding, i.e., variations between 1.33 and 1.34, we can affirm that the most suitable height for our component is 450 nm, since a long penetration depth of the evanescent wave and the largest possible $\Delta n_{eff}$ for the small variation induced in the refractive index of the cladding, is guaranteed.

**Figure 6:**
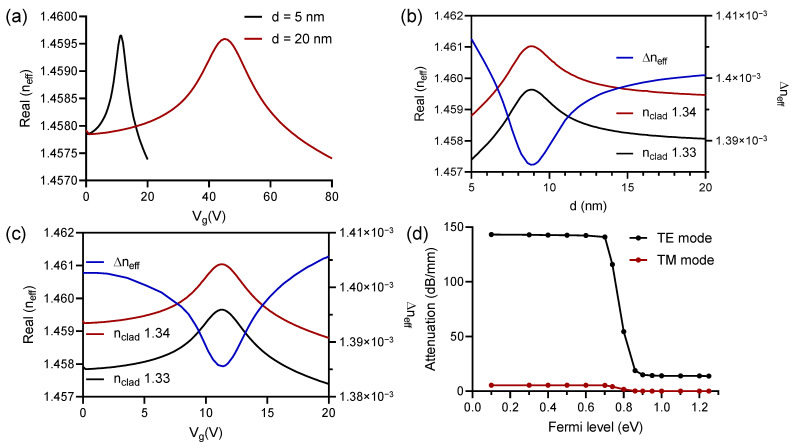
(**a**) Real part of the effective refractive index as a function of the Vg for 5 nm and 20 nm thickness in the dielectric between the two monolayers of graphene. (**b**) Analysis at $V_{g}=20$ V of the real part of the effective refractive index as a function of the *d* variation, for cladding values at 1.33 and 1.34, and the $\Delta n_{eff}$ as a function of the reference arm. (**c**) Analysis at d=5 nm of the real part of the effective refractive index as a function of the $V_{g}$ variation, for cladding values at 1.33 and 1.34, and the $\Delta n_{eff}$ as a function of the reference arm. (**d**) Attenuation as a function of the Fermi level variation, for TE and TM propagation modes.

Previously, the impact of the capacitive structure in the propagated optical mode was observed, in this section it is necessary to define the thickness *d* of dielectric layer of the capacitor in order to tune the Fermi energy of the graphene with low power, maximizing the sensitivity of the proposed sensor. As described by Equations (3) and (4), $V_{g}$ is directly proportional to η, so in order to reduce the applied voltage, smaller thicknesses are desired, as shown in Figure 6.

By reducing *d* from 20 nm to 5 nm thickness, it is possible to observe a shift in the maximum value reached by the effective refractive index from 44 V to 11 V, observing a reduction in the power required to tune the optical absorption of the graphene in approximately 40 V. This can be seen in Figure 6a. Additionally and according to Figure 6b, lower thicknesses can provide also an increase in the refractive index variation, demonstrating the highest $\Delta n_{eff}$ at $1.405\times 10^{-3}$ for d=5 nm. For this value of thickness it was obtained a surface charge density of 1.1fF/m.

Similarly, we analyzed the biosensor response as a function of the $V_{g}$ variation in Figure 6c. We demonstrated that when the real part of the effective refractive index is maximum, the $\Delta n_{eff}$ calculated for small variations of the refractive index in the cladding, i.e., variations between 1.33 and 1.34 is minimum. In addition, we observed that increasing the $V_{g}$ further than 11 V the $\Delta n_{eff}$ also increase. A $V_{g}$ at 20 V guarantees a good variation of the $\Delta n_{eff}$ for biosensing applications, and banish the possibility of polymeric deformation due to the high temperatures that can arise from the implementation of high voltage levels.

We reported, by simulations, that tuning the Fermi energy level of the graphene enables the electro-optical modification in the sensor, according to Equation (3). The absorption of TE mode change from 143.14 dB/mm at 0.1 eV to 13.83 dB/mm for 1.25 eV such values demonstrate the high interaction of the evanescent wave in the sensing area contributing to optimize the sensibility of the sensor, as presented in Figure 6d.

## 3. Results and Discussion

To evaluate the performance of the proposed sensor, we conducted simulations in frequency domain, evaluating the complete layout using Finite Element Method (FEM) in COMSOL Multiphysics.

The effective refractive indices calculated in the previous section for the corresponding sensing area and for the complementary section of the MZI, were incorporated in this simulation system, considering the variation that the refractive index presents between 400 nm and 800 nm wavelengths.

**Figure 7:**
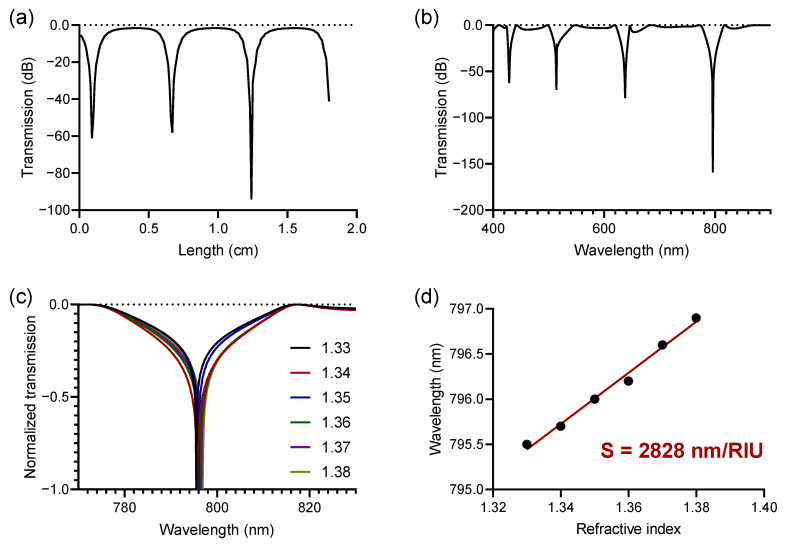
Propagation analysis in the proposed MZI biosensor, based on double monolayer of graphene capacitor. (**a**) Transmission response where the phase shift is evaluated as a function of the variation of the length of the sensing area ($L_{sens}$) at 800 nm wavelength and 1.33 refractive index in the cladding (**b**) Transmission response where the phase shift is evaluated as a function of the wavelength variation between 400 nm and 900 nm, for 1.24 cm length in the sensing area. (**c**) Close-view of the redshifted normalized transmission in the region where the maximum phase shift occurs, for refractive indices between 1.33 and 1.38 with a step of 0.01 RIU. (**d**) Sensitivity obtained through a linear regression calculated on the simulations carried out in (**c**).

In order to optimize the total length of the sensing area ($L_{sens}$), we simulated the transmission response of the sensor as a function of the $L_{sens}$, evaluating the spectral response for $L_{sens}$ ranging from 100 μm up to 1.8 cm length. In Figure 7a, it is possible to appreciate the maximum phase shift when $L_{sens}$ is 1.24 cm, considering 1.33 refractive index in the cladding. In accordance with the above, in Figure 7b the phase shift was verified as a function of the variation of the wavelength between 400 nm and 900 nm, observing an excellent result at 800 nm wavelength, in accordance with the results discussed in the previous section.

Setting the total length of the sensing area at 1.24 cm, and propagation wavelength at 800 nm, we analyzed the perceived transmittance at the output of the component for different values of refractive index in the cladding, varying from 1.33 up to 1.38 with a step of 0.01 RIU. According to Figure 7c, it is possible to observe that despite maintaining the phase shift at close wavelengths, the resonance wavelength shows a redshift as a function of the increase in the refractive index in the sensing area. This can mean that small variations in bulk can be detected with high precision due to the high sensitivity demonstrated, i.e., 2828 nm/RIU. The calculation of the sensitivity achieved can be seen in Figure 7d, where by applying a linear regression to the calculated points, it was possible to obtain from the curve slope a sensitivity of 2828 nm/RIU for the simulated device. As Table 1 shows, our sensor obtained improvements in relation to the sensitivity of similar biosensors, even in relation to silicon-based components.

Finally, we carry out sensitivity studies on the surface, where a biolayer with a refractive index of 1.45 and 10 nm thick [32] was added to our model, in order to observe the performance of the polymeric transducer. The result observed for the sensitivity on the surface, i.e., 2021.85 nm/RIU at $V_{g}$ = 20 V, demonstrate consistency with those presented in the bulk device, suffering a reduction of only 28% in comparison with the latter, which is the main differential of this technology. This decrease in sensitivity is due to the fact that the presence of the biological layer prevents a greater penetration depth of the evanescent field in the sensing area, reducing the intensity of the external field and consequently reducing the efficiency of the proposed sensor. However, and against the forecasts, the performance of our transducer shows a decrease much less than that presented in other types of devices, where it is observed that the reduction between the sensitivity obtained in bulk and surface, is around 40% for devices in silicon technology [32,46] and greater than 60% in polymer technology [32], limiting them in terms of Limit of Detection (LoD). This good performance is due to the interaction induced between the propagated optical mode and the electric field generated by the embedded capacitor, which shows significant improvements compared to previously proposed sensors.

## 4. Conclusions

In summary, a MZI sensor based on embedded double-monolayer graphene capacitor in polymer technology is presented and demonstrated. The embedded capacitive structure was modeled, guaranteeing the tuning of the Fermi energy level, aiming to reduce the optical absorption of the graphene, and simultaneously guaranteeing an increase in the evanescent field in the sensing area, due to the interaction induced between the optical mode propagated in the polymer waveguide and the electric field in the proposed capacitive structure. The optimization of the sensing area in the MZI allows increasing the sensitivity that can be achieved, because it allows strong confinement of the light in the general waveguide and a long penetration depth of the evanescent field in the sensing area. Our device was evaluated as a biosensor, presenting a bulk sensitivity of 2828 nm/RIU and a surface sensitivity of 2021.85 nm/RIU, both with a power $V_{g}$ = 20 V, demonstrating a sensitivity greater than that achieved by similar devices available from experimental results in the scientific literature [41,42,43,44,45,46,47,48,49,50,51,52].

Due to the high sensitivity demonstrated and the materials used, these devices represent an important advance in obtaining Label-Free disposable optical biosensors for Lab on a Chip (LoC) platform, for the early detection of genetic and infectious diseases.

## Figures and Tables

**Table 1:** Comparison between the proposed MZI biosensor based on double-layer graphene capacitor, with other optical biosensors presented to the scientific community, based on polymer or silicon technology.

Sensor Type	Waveguide Material	Mode	Sensitivity
Ridge waveguide	Polymer—PSQ-Ls	TE	49.75 nm/RIU [41]
Grating-based waveguide	Polymer—FSU-8	TE	1606.2 nm/RIU [42]
Microring resonator	Polymer—ZPU13-430	TE	200 nm/RIU [43]
MZI	Silicon Nitride	TE	1864π/RIU [44]
Slot Waveguide	Silicon Nitride	TE	1730(2π)/RIU [45]
MZI	Silicon	TE	740 nm/RIU [46]
MZI	Silicon	TM	460(2π)/RIU [47]
MZI	Silicon	TE	300(2π)/RIU [48]
Photonic crystal	Silicon	TM	425 nm/RIU [49]
Microring resonator	Silicon	TM	579.5 nm/RIU [50]
Photonic crystal	Silicon	TE	404.11 nm/RIU [51]
Photonic crystal	Silicon	TE	300 nm/RIU [52]
MZI biosensor based on double-layer graphene capacitor	Polymer—SU-8	TE	2828 nm/RIU [This work]

## Data Availability

The data presented in this study are available on request from the corresponding author.
